# Censoring Distances Based on Labeled Cortical Distance Maps in Cortical Morphometry

**DOI:** 10.3389/fneur.2013.00155

**Published:** 2013-10-14

**Authors:** Elvan Ceyhan, Tomoyuki Nishino, Dimitrios Alexopolous, Richard D. Todd, Kelly N. Botteron, Michael I. Miller, J. Tilak Ratnanather

**Affiliations:** ^1^Department of Mathematics, Koç University, Istanbul, Turkey; ^2^Department of Psychiatry, School of Medicine, Washington University, St. Louis, MO, USA; ^3^Department of Genetics, School of Medicine, Washington University, St. Louis, MO, USA; ^4^Department of Radiology, School of Medicine, Washington University, St. Louis, MO, USA; ^5^Center for Imaging Science, Johns Hopkins University, Baltimore, MD, USA; ^6^Institute for Computational Medicine, Johns Hopkins University, Baltimore, MD, USA; ^7^Department of Biomedical Engineering, Johns Hopkins University, Baltimore, MD, USA

**Keywords:** computational anatomy, depression, morphometry, pairwise comparisons, censored distance, ventral medial prefrontal cortex

## Abstract

It has been demonstrated that shape differences in cortical structures may be manifested in neuropsychiatric disorders. Such morphometric differences can be measured by labeled cortical distance mapping (LCDM) which characterizes the morphometry of the laminar cortical mantle of cortical structures. LCDM data consist of signed/labeled distances of gray matter (GM) voxels with respect to GM/white matter (WM) surface. Volumes and other summary measures for each subject and the pooled distances can help determine the morphometric differences between diagnostic groups, however they do not reveal all the morphometric information contained in LCDM distances. To extract more information from LCDM data, censoring of the pooled distances is introduced for each diagnostic group where the range of LCDM distances is partitioned at a fixed increment size; and at each censoring step, the distances not exceeding the censoring distance are kept. Censored LCDM distances inherit the advantages of the pooled distances but also provide information about the location of morphometric differences which cannot be obtained from the pooled distances. However, at each step, the censored distances aggregate, which might confound the results. The influence of data aggregation is investigated with an extensive Monte Carlo simulation analysis and it is demonstrated that this influence is negligible. As an illustrative example, GM of ventral medial prefrontal cortices (VMPFCs) of subjects with major depressive disorder (MDD), subjects at high risk (HR) of MDD, and healthy control (Ctrl) subjects are used. A significant reduction in laminar thickness of the VMPFC in MDD and HR subjects is observed compared to Ctrl subjects. Moreover, the GM LCDM distances (i.e., locations with respect to the GM/WM surface) for which these differences start to occur are determined. The methodology is also applicable to LCDM-based morphometric measures of other cortical structures affected by disease.

## Introduction

Recent advances in high resolution magnetic resonance imaging (MRI) technology and developments in computational anatomy (CA) methods [see, e.g., Ref. (1–6)] have resulted in a substantial increase in understanding of the laminar structure of the neocortex. There are two analytical approaches for studying cortical thickness: global or regional level. Whole brain approaches rely on atlases and mapping to flat or spherical templates. Thus these methods are sensitive to the choice of atlases (7) and distortion in mapping to the templates (8). Most region of interest (ROI) approaches have stemmed generally from whole brain parcellation and can thus be influenced by whole brain data rather than local, i.e., ROI image data. These problems can be overcome or minimized by a ROI focused approach which analyzes the subvolume encompassing the cortical ROI which is the basis of the Labeled Cortical Distance Mapping (LCDM) approach used here.

The ROI approach requires precise definitions of anatomical boundaries which can be compounded by anatomical variability in development or degeneration. This can be overcome by viewing the ROI as a laminar mantle composing of gray matter (GM) voxels and a GM/white matter (WM) cortical surface (3). LCDM data are distances of labeled GM voxels with respect to the GM/WM cortical surface, and so quantize and characterize the morphometry of the laminar cortical mantle. Here “morphometry” has two components, the structural formation (like surface and form of the tissue) and scale or size (like volume and surface area). Thus, morphometry refers to all aspects of laminar shape, where “shape” refers to the surface structure, and “size” refers to the scale of the tissue in question.

The LCDM approach has been applied in clinical neuroimaging studies of the cingulate in subjects with Alzheimer’s disease (2) and schizophrenia (1, 6, 9), the prefrontal cortex in subjects with major depressive disorder (MDD) (10) and schizophrenia (11), the parahippocampal gyrus in subjects with schizophrenia (12), the occipital cortex in visual attention (4, 13), area 46 of the frontal cortex in fetal irradiated macaques (14), and entorhinal cortex in normal aging controls and in subjects with mild cognitive impairment (15). Finally, our observation of variable cortical thickness in the left PT in three groups of age-matched and gender-matched controls and patients with schizophrenia and bipolar disorder (5) is consistent with post-mortem analysis (16). The approach has also been extended to deal with deeply buried sulci by modeling image intensity stochastically based on the normal distance where the model includes cortical thickness as one of the parameters (17); others have similarly adapted LCDMs (18, 19).

The LCDM approach is similar to the voxel-based cortical thickness (VBCT) method (20) where each voxel in the GM has a thickness value associated with it, but our analysis of these voxel-based thickness values is different. In VBCT, cortical thickness values are compared on a voxel-by-voxel basis as in SPM (21), while our analysis of LCDM distances allows us for example to first pool (i.e., merge) the distance values for each diagnostic group, and perform the comparisons on the overall distance (or thickness) level, rather than the voxel level for each individual. But it has been shown that LCDMs are comparable to other methods for computing cortical thickness (22) and that LCDM profiles for whole brains are similar in shape (20, 23).

In our LCDM approach, the surface between GM/WM is determined, and then distance of each voxel to this surface is computed (2, 3). Previously, we have discussed the analysis of these pooled distances for the overall comparison of morphometric differences due to depression (10).

Analysis of volumes (in cubic millimeter), of descriptive measures (i.e., summary statistics) of pooled distances, and of the pooled distances yield “rough” comparisons of cortical ROIs between groups, in the sense that, if significant, a comparison indicates global morphometric (shape and/or size) differences in cortical ROIs between groups (10, 24). But they do not reveal where (e.g., at which distance from GM/WM surface) these differences occur. As the LCDM distances measure the distance from GM voxel centers to GM/WM surface, they carry more than just shape/size information. This suggests that, properly used, LCDM distances may also provide at which distance GM in the cortical ROI differ between groups, thereby providing additional information about the underlying nature of the difference associated with the disease.

Abnormalities have been demonstrated in structure and function of specific regions of the prefrontal cortex associated with MDD (25, 26). Previous structural imaging studies have largely focused on adult onset MDD, while only a few have focused on early onset MDD. Structural deficits in a subregion of the Ventral Medial Prefrontal Cortices (VMPFCs), i.e., subgenual prefrontal cortex, have also been associated with early onset of MDD (27–32). LCDM data for the VMPFC has been analyzed in detail (10, 24). Here, the data based on a twin design neuroimaging study contained three diagnostic groups, namely, MDD, being at high risk (HR) for MDD, and the control (Ctrl) group. Morphometric summary measures such as mean, median, variance, etc. of the LCDM distances and volumes were analyzed (24), but these summary statistics failed to detect differences between MDD and healthy subjects. Since such measures were oversimplifying the vast amount of information in LCDM data, pooling of the LCDM distances by diagnostic group, rather than subsampling, was introduced so as to detect morphometric differences with a higher sensitivity (10). In pooled LCDM distances, the entire LCDM data set was used, and the validity of the underlying assumptions for the tests was investigated. Significant morphometric differences in VMPFC were observed associated with MDD or being at HR for MDD.

In this article, we propose censoring of LCDM distances which may provide more information about the distribution of GM voxels. In censoring, we partition the range of LCDM distances at a particular increment size, and at each increment, we only keep LCDM distances not exceeding the corresponding censoring distance relative to the GM/WM surface. As an illustrative example, we use the same LCDM data for VMPFC (10, 24) so as to demonstrate the benefits of censoring LCDM distances compared to pooled LCDM distances. Censored LCDM distances inherit the advantages of the pooled distances (such as robustness to assumption violations and sensitivity to morphometric differences due to a disease) and also provide information on the laterality and location of changes associated with the disease in question. In particular, by using the censored distances, one can determine where significant differences in GM of VMPFC occur related to MDD or HR in terms of distance to the GM/WM surface. By Monte Carlo simulations, we demonstrate that comparison of censored distances between diagnostic groups is robust to the violations of the underlying assumptions such as within sample independence and normality (i.e., Gaussianity). Furthermore, at each censoring step distances less than or equal to the corresponding censoring distance aggregate, and this might confound the results of the analysis. Our extensive Monte Carlo study also indicates that such an aggregation effect is negligible for censored distances. Additionally, censored distances are very sensitive to indicate differences as a function of distance from the GM/WM surface.

We describe the example data and its acquisition in Section “Data Description and Acquisition,” censoring methods in Section “Censoring LCDM Distances,” statistical methodology in Section “Statistical Methodology,” analyze the censored distances in Section “Results,” and investigate the influence of aggregation of censored distances and assumption violations with an extensive Monte Carlo simulation study also in Section “Results,” and provide discussion and conclusions in Section “Discussion.”

## Materials and Methods

### Data description and acquisition

The MRI tools and methodology to prepare VMPFC to measure LCDM distances, and the measurement process of LCDMs have been described in detail (10). Here we provide them again for the sake of completeness. In order to study cortical changes in the VMPFC associated with MDD, a cohort of 34 right-handed young female twin pairs between the ages of 15 and 24 years old were obtained from the Missouri Twin Registry. The inclusion criteria for affected twin pairs were the DSM-IV criteria for MDD being greater than duration of 4 weeks and the onset prior to age 16. Control twin pairs had no personal or first degree of family history of MDD. Both monozygotic and dizygotic twin pairs were included; 14 pairs of twins were controls (Ctrl) and 20 pairs had one twin affected with MDD, their co-twins were designated as the HR group. The Washington University School of Medicine Human Studies Committee approved the study that collected the subjects who all gave written informed assent (if under 18 years old) or consent (18 years old or older) for participation in the study. Parents of subjects under 18 years of age also gave written informed consent. Three high resolution T1-weighted MPRAGE magnetic resonance scans of each subject in this population were acquired using a Siemens scanner with 1 mm^3^ isotropic resolution (sagittal acquisition, repetition time [TR] = 10 ms; echo time [TE] = 4 ms; time to inversion [TI]/time delay [TD] = 20/0; flip angle = 10°; slab = 160 mm; 160 partitions; 256 × 256 matrix; field of view [FOV] = 256; 4 signal averages; total scanning time: 26 min 55 s). Images were then averaged, corrected for intensity inhomogeneity and interpolated to 0.5 mm × 0.5 mm × 0.5 mm isotropic voxels. Following Ref. (33), a ROI comprising the VMPFC stripped of the basal ganglia, eyes, sinus, cavity, was defined manually and segmented into GM, WM, and cerebrospinal fluid (CSF) by Bayesian segmentation using the expectation maximization algorithm (34). A triangulated representation of the cortex at the GM/WM boundary was generated using isocontouring algorithms (34).

Partial volume, i.e., voxels that share mixtures were resolved via a Neyman–Pearson recalibration of the segmentation based on a training set (33). The threshold between GM and WM was used to generate a triangulated isosurface via the marching tetrahedra algorithm i.e., the mesh is dense. Validation with several VMPFC subvolumes yielded misclassification errors of 0.05–0.10 (*n* = 5) for the segmentation and sub-voxel accuracy of the isosurface with 50% of the vertices within 0.12–0.28 mm (*n* = 14) from semi-automated contours (33).

**Figure 1:**
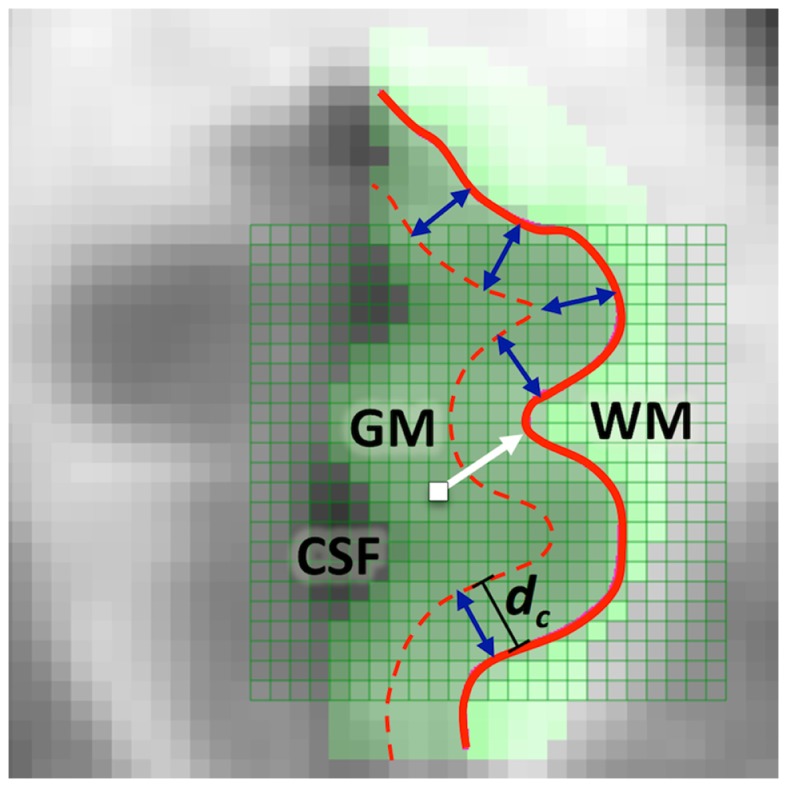
**A two-dimensional illustration of LCDM distance (i.e., the normal distance) from a GM voxel to the GM/WM surface (thick white arrow) and of the censoring procedure with censoring distance *d_c_* (blue double arrows)**. At this censoring step, the GM voxels whose centroids closer to the GM/WM surface than *d_c_* are retained.

Labeled cortical distance mapping is generated by measuring the distance from the GM/WM surface to the center of mass of each voxel. More specifically, first, the ROI subvolume is partitioned by a regular lattice of voxels of specific size *h*, denoted *V*(*h*). Every voxel is labeled by tissue type as GM, WM, or CSF [see, e.g., Ref. (3, 34)]. For every GM voxel in the ROI, the distance from the centroid of the voxel to the closest point on GM/WM surface is computed. Let *S*(Δ) be the triangulated graph representing the GM/WM surface. An LCDM distance is a set distance function *d*: ν*_i_* ∈ *V*(*h*) → (ν*_i_*, *S*(Δ)), the distance between the centroid of voxel ν*_i_* and the set *S*(Δ); that is, it is the distance from the center of the voxel to the closest vertex on the surface. More precisely,

(1) $$D_{i}:=d\left(C_{M}\left(\nu_{i}\right),\;S\left(\Delta \right)\right)=\underset{s\in S\left(\Delta \right)}{\min}\;{∥C_{M}\left(\nu_{i}\right)-s∥}_{2}$$

where *C_M_*(⋅) stands for center of mass (or centroid), and ||⋅||_2_ is the usual *L*_2_ – norm. Computation of the LCDM distance for a GM voxel is illustrated in Figure 1 (with a thick white arrow). We use a signed (or labeled) distance to indicate the location of each voxel with respect to the GM/WM surface. As GM tissue comprises most of the cortex, and by construction, while most of GM distances are positive, negative distances for some GM close to the GM/WM boundary are possible by construction, because the surface is constructed in such a way that a surface is always intersecting voxels, i.e., partial volume. So some appropriately labeled GM voxels may fall on a side of surface that they should not belong to. However, these mislabeled voxels constitute a small proportion of all voxels and do not affect the overall analysis. Reliability of LCDMs is dependent on GM segmentation and reconstruction of GM/WM surfaces which has been validated for several cortical structures including VMPFC (33), cingulate cortex (6, 35), and planum temporale (36). Condensing to a single distance value for each vertex on the surface is the next logical step in extending LCDM. This is called Local LCDM or LLCDM and is useful in comparing thickness across multiple subjects for a cortical structure [see Ref. (5, 9)].

**Figure 2:**
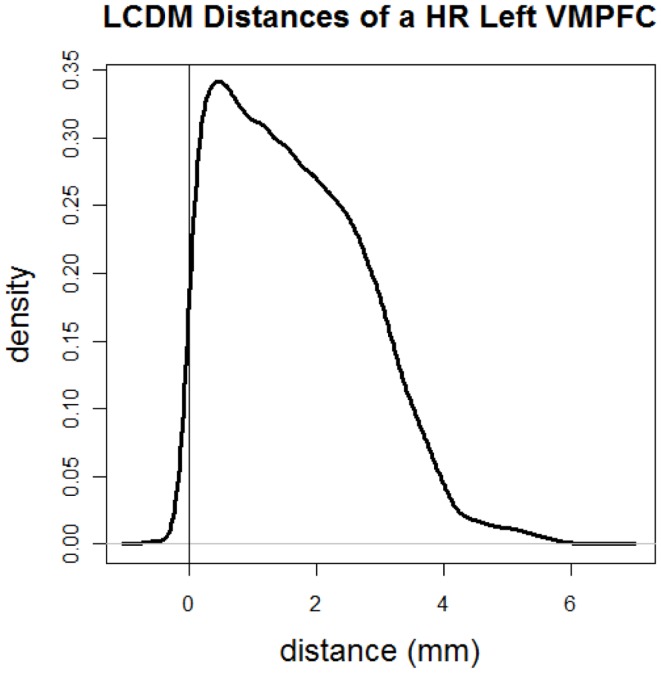
**Kernel density estimate of directed (i.e., signed) LCDM distances of GM voxels for a sample cortical structure of interest**. More specifically distances are for the GM of the left VMPFC of a HR subject.

Figure 2 illustrates the kernel density estimate of LCDM distances of GM voxels of a typical cortical structure of interest. In this cortical structure most of GM distances are positive. If two LCDM distance sets are different (with everything else being same), one can safely deduce that the corresponding VMPFCs have different morphometric structures. Thus, LCDM may serve as a tool to analyze and/or compare the morphometry (shape and size) of cortical tissues in brain. However the converse is not necessarily true. Two tissues with different morphometry might have exactly the same LCDM distribution. Hence, LCDM distances do not entirely characterize the morphometry of the ROI, however, when all the distances from the diagnostic groups are merged, this problem gets less severe. In fact, our goal is not reconstruct the ROI given the LCDM distances, but to detect morphometric differences based on LCDM distances. The significant differences in LCDM distances would imply significant morphometric differences, but insignificant differences would only imply lack of evidence for morphometric differences as in the Neyman–Pearson hypothesis testing paradigm (37).

Let *D^L^*(*D^R^*) be the set of LCDM distances for the left (right) ROI. Then $D^{L}=\{D_{ijk}^{L},\;i=1,2,3,\;j=1,2,\;\ldots ,\;n_{i},\;k=1,2,\;\ldots ,\;K_{ij}\}$ where $D_{ijk}^{L}$ is the LCDM distance for the *k*th voxel in GM of left VMPFC of subject *j* in group *i* (with *i* = 1 for MDD, *i* = 2 for HR, and *i* = 3 for Ctrl). So, we have *n*_1_ = 20, *n*_2_ = 20, and *n*_3_ = 28. Right VMPFC distances *D^R^* are denoted similarly as $D^{R}=\left\{D_{ijk}^{R},\;i=1,\;2,\;3,\;j=1,\;2,\;\ldots \;,\;n_{i},\;k=1,\;2,\;\ldots \;,\;K_{ij}\right\}$. We only retain LCDM distances between −0.5 and 5.5 mm based on prior anatomical knowledge [e.g., Ref. (38)], so as to avoid voxels potentially mislabeled as GM. By doing so only a negligible portion (≤0.20%) of the distances are removed from further analysis. Keeping these distances could have confounded our results, especially for very small and very larger distances.

### Censoring LCDM distances

To obtain information on the location of morphometric differences measured as distance to the GM/WM surface, we propose the following procedure, which is called *censoring of LCDM distances*. In censoring, we first determine a set of threshold distances, and at each step keep the distances for each subject up to the threshold value, pool the retained distances for each group and then perform various analysis on the retained distances. More specifically, we first partition the set of LCDM distances into bins of size δ, then we have ⌊*d*_max_/δ⌋many bins where *d*_max_ is the largest LCDM distance value in $D^{L}⋃D^{R}$ and ⌊*s*⌋ stands for the floor of *s*, i.e., largest integer less than or equal to *s*. In order to construct LCDM censored distances, we only retain distances less than or equal to the specified distance value. That is, at *k*th censoring step, we only consider the voxels whose LCDM distance is less than or equal to γ*_k_*_,δ_ = *k*δ. Thus we only consider the layer of the cortex with distance of γ*_k_*_,δ_ or less from the GM/WM surface. An illustration of censoring distances at the threshold distance *d*_c_ for a subject is provided in Figure 1, where the GM voxels whose centroids are closer to the GM/WM surface than *d_c_* are retained at this particular censoring step. These distances are the censored LCDM distances, which, for left VMPFCs, are denoted as,

(2) $$C_{d}^{L}\left(k,\delta \right):=\left\{d\in D^{L}⋂\left[-0.5,\;k\delta \right]\right\}=\left\{d\in D^{L}:d\leq k\delta \right\}$$

and for group *i* in left VMPFCs,

(3) $$C_{d,i}^{L}\left(k,\delta \right):=\left\{d\in D_{i}^{L}:d\leq k\delta \right\}$$

for *i* = 1, 2, 3 (i.e., for groups MDD, HR, and Ctrl, respectively). Censored LCDM distances for right VMPFCs are denoted similarly as $C_{d}^{R}\left(k,\delta \right)$ and as $C_{d,\;i}^{R}\left(k,\;\delta \right)$ for group *i*, respectively. This procedure is called *censoring*, because distances are measured for voxels, if the centroids of the voxels are closer to the GM/WM surface than a threshold, and the distances for the remaining voxels are discarded. By censoring LCDM distances, we partition the VMPFCs with respect to distance from GM/WM surface; thereby can obtain more detailed and more localized morphometrics of the VMPFCs compared to the pooled LCDM analysis. For example, if analysis of censored distances yields a significant result at step *k*, it would indicate significant morphometric differences between diagnostic groups at GM distance of *k*δ millimeter. If significant differences are observed at all censoring steps between *k* and *l*, then this would mean that significant morphometric differences occur for GM distance values between *k*δ and *l*δ millimeter. With pooled LCDM distance analysis, we would only know presence or absence of any morphometric differences between diagnostic groups without any indication of its whereabouts. However, the censoring of LCDM distances is suggestive of which distance (from the GM/WM surface) the significant differences in morphometry occur. Yet, censoring of LCDM distances indicates the differences along the single dimension that relates to the normal distance from the GM/WM surface (i.e., thickness of layers of the cortical mantle), but are not spatially localized along three dimensions. Although this potentially reduces the utility of the method, it is a substantial improvement compared to the pooled LCDM analysis and prospective research along this direction could provide more localized three dimensional information.

In the following sections, we use *d*_max_ = 5.5 mm and we pick δ = 0.01 mm, hence *k* = 0, 1, 2, …, 550 and γ*_k_*_,δ_ = 0.00, 0.01, 0.02, …, 5.50 mm. Due to the confounding influence of mislabeled GM voxels close to the GM/WM surface, censoring distances in [1, 5.5] mm provide more reliable results. Note also that for γ*_k_*_,δ_ = 5.5 mm, i.e., at the last censoring step, the censored distance analysis is identical to the pooled distance analysis provided (10).

### Statistical methodology

For a specific subject, the LCDM distances for neighboring voxels are correlated; hence there is an inherent spatial dependence between LCDM distances at the individual level. Pooling and censoring do not remove this dependence; on the contrary, they ignore the subject information but only take diagnostic group information into account. We previously considered Kruskal–Wallis (K–W) and ANOVA *F-*tests for multi-group comparisons and Wilcoxon rank sum test and Welch’s *t-*test for pairwise comparisons of pooled distances (10). Furthermore, Kolmogorov–Smirnov (K–S) test was employed for distributional comparisons [see Ref. (39) for information on these tests]. However these tests only detect global morphometric differences but do not provide where in the ROI (e.g., VMPFCs) these differences occur. It has been demonstrated that the influence of these assumption violations is negligible (10).

We introduce censoring of LCDM distances to find out where (i.e., at which distance value) the significant differences occur. For left (and right) censored distance comparisons, at each censoring step, we apply K–W test for testing the equality of the distributions for all (three) groups and ANOVA *F-*test with and without assuming homogeneity of the variances of the distances for testing the equality of the means of the distances. These tests are used to detect possible differences between groups in these censored distances. If K–W test yields a significant *p-*value at a censoring distance value, then the morphometry is different for at least two of the groups and this difference starts to occur at this censoring distance value. To find out which pairs of groups exhibit significant morphometric differences at this distance, we use pairwise Wilcoxon rank sum test to compare the pairs of the groups. Similarly, if one of the ANOVA *F*-tests is significant, then we use pairwise *t*-test to compare the pairs of the groups. We perform similar analyses for right censored LCDM distances. See, e.g., Ref. (39) for more detail on the tests.

When applied on censored LCDM distances, K–W test, and Wilcoxon tests may provide at which distance the distributional differences occur, and ANOVA *F*-tests and Welch’s *t*-tests might provide at which distance the mean LCDM distances start to differ. However, K–S test might be misleading when applied to censored distances, since it will indicate the distance where the first significant difference occurs, but by construction, the test will tend to yield the same (or more significant) *p*-values at subsequent censoring steps.

For each of the above tests, if the tests start to be significant at a certain censoring distance, say *d*_1_ and stays significant for subsequent steps up to distance *d*_2_, then the morphometric differences in the GM tissue start to be detectable by LCDM distances at voxels whose distance is at or larger than *d*_1_ and the significant morphometric difference persists up to distance *d*_2_. Hence the importance of the censoring of the LCDM distances: it provides not only significant morphometric differences, but also where (i.e., at which distance value) the differences are located (with respect to the GM/WM surface).

## Results

### Analysis of censored LCFM distances of VMPFCs

We have pooled the LCDM distances of subjects in the same group and kept distances between [−0.5, 5.5] mm and at each censoring distance, γ*_k_*_,δ_, we have the distance values in [−0.5, γ*_k_*_,δ_] mm. These censored distances convey shape/size information at the specified γ*_k_*_,δ_ value, i.e., at distance of γ*_k_*_,δ_ or less from the GM/WM surface. At each censoring step *k*, the distribution of the censored distances (hence distribution of pooled distances) is severely non-normal based on Lilliefor’s test of normality (all *p-*values are virtually zero).

Among the underlying assumptions of the parametric tests (ANOVA *F*-tests and *t*-tests), within sample independence and normality (Gaussianity) of LCDM distances are violated and for the morphometric tests (K–W and Wilcoxon rank sum tests), within sample independence is violated. However the violation of these assumptions for the pooled LCDM distances was shown to be negligible (10). The censored LCDM distances inherit this robustness property of the pooled distances (as the censoring is performed on the pooled distances). Although more assumptions are violated for the parametric tests, we still use them, since both parametric and non-parametric tests are not influenced by these violations (10). Furthermore, parametric tests are more sensitive against the alternatives that influence the means, while non-parametric tests are more sensitive against the alternatives that influence the ranking (i.e., ordering) of the distances. Due to the confounding effect of mislabeled voxels, we only consider the censoring distance analysis for [1.0, 5.5] mm, as the analysis for this range will be more reliable. This cautionary measure is in effect for this sample data set, and if the problem of mislabeled voxels is minute or sufficiently small, one could consider the whole range of distances (i.e., [−0.5, 5.5] mm).

#### Multi-group comparisons by censored LCDM distances

Kruskal–Wallis test and ANOVA *F*-tests yield significant differences between LCDMs of the three groups (Ctrl, HR, MDD) (*p* < 0.0001 for each multi-group test). Hence there are significant morphometric differences (in each of left and right VMPFCs) in at least two of the diagnostic groups in question. Then, we test for group differences in censored LCDM distances to see at which distance value the significant differences start to occur. The null hypothesis for K–W test for left censored distances is the equality of the distributions of the left censored distances, namely,

(4) $$H_{o}:F_{1}^{L}\left(k,\delta \right)=F_{2}^{L}\left(k,\delta \right)=F_{3}^{L}\left(k,\delta \right)$$

where $F_{i}^{L}\left(k,\delta \right)$ is the distribution of left censored LCDM distances at censoring step *k* with increment size δ for group *i* = 1, 2, 3 (i.e., MDD, HR, and Ctrl, respectively). The null hypothesis for ANOVA *F*-test [with or without homogeneity of variances (HOV)] for left censored distances is the equality of the means of the censored distances, namely,

(5) $$H_{o}:\mu_{1}^{L}\left(k,\;\delta \right)=\mu_{2}^{L}\left(k,\;\delta \right)=\mu_{3}^{L}\left(k,\;\delta \right)$$

where $\mu_{i}^{L}\left(k,\;\delta \right)$ is the mean of left censored LCDM distances at censoring step *k* with increment size δ for group *i* = 1, 2, 3. The null hypotheses for the right censored LCDM distances are similar with *L* being replaced with *R*.

**Figure 3:**
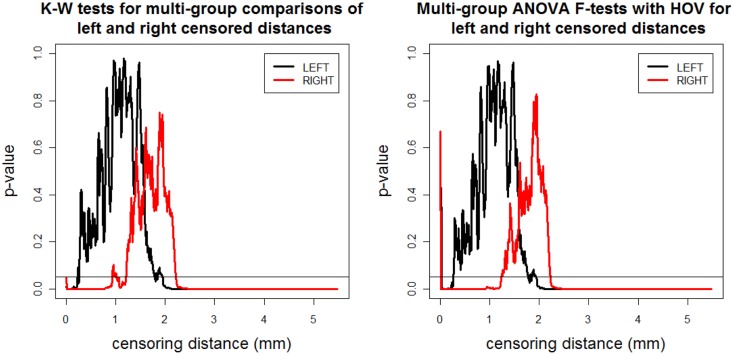
**The *p*-values versus censoring distance values (millimeter) for multi-group K–W test (left) and ANOVA *F*-test with HOV (right) for LCDM distances for left (thick black solid line) and right (thin red solid line) VMPFCs**. Horizontal lines are located at 0.05 to indicate the threshold values for significance.

We record the *p-*values for K–W test and ANOVA *F*-test with HOV and plot them against censoring distance (i.e., γ*_k_*_,δ_) values in Figure 3 where the horizontal line is located at 0.05. When the *p*-values fall below the nominal significance level of 0.05, they are deemed to be significant. Observe that the plots for K–W test and ANOVA *F*-test with HOV are very similar and so is the plot for ANOVA *F*-test without HOV (hence not presented). The alternative for K–W and ANOVA *F*-tests does not have a direction for three or more groups. So a *p-*value <0.05 for K–W test (ANOVA *F*-test with or without HOV) implies that for at least two groups, the distributions (means) of the distances less than or equal to γ*_k_*_,δ_ are different. Based on K–W test (ANOVA *F*-test with HOV); we observe that the differences between distributions (means) of left and right censored distances start to occur at about the same distance value. The distributions and means of the distances are significantly different for at least two of the groups for distance values of 2.00 mm or larger for left VMPFCs, and 2.20 mm or larger for right VMPFCs. Significant differences occur for right VMPFC distances between 0 and 1.2 mm as well, however due to confounding nature of negative VMPFC distances, this result is reliable for the range of 1–1.2 mm. This implies that there are significant morphometric differences due to depression at distance values of 2.00 mm or larger in GM of left VMPFCs and around 1–1.2 and 2.20 mm or larger in GM of right VMPFCs.

#### Pairwise comparisons by censored LCDM distances

Pairwise comparisons of the LCDM distances for the diagnostic groups indicate that LCDM distances for MDD and HR groups are not significantly different for both left and right VMPFCs (*p*-values are 0.6043 and 0.1553, respectively). The LCDM distances for both MDD and HR left and right VMPFCs are significantly smaller than those counterparts of Ctrl left and right VMPFCs (*p* < 0.0001 for all). Hence significant reduction in laminar thickness is observed in VMPFCs associated with MDD, but the same trend is also observed associated with being at HR for MDD as well (10).

We also found at which distance values the distributions of the censored distances are different between groups. The next question of interest is which pairs of groups are different at each distance value. Along this line, we perform pairwise comparisons of censored distance values at each censoring step *k*. For left and right VMPFC distances, at each censoring distance, γ*_k_*_,δ_, we test for each pair of groups by Wilcoxon rank sum test for both less than and greater than alternatives, and record the corresponding *p-*values. The simultaneous hypotheses for Wilcoxon tests for left censored LCDM distances are,

(6) $$\begin{array}{llll} & H_{o}:F_{1}^{L}\left(k,\;\delta \right)=F_{2}^{L}\left(k,\;\delta \right)\;\mathrm{and}\;F_{1}^{L}\left(k,\;\delta \right)=F_{3}^{L}\left(k,\;\delta \right)\;\mathrm{and}\; & & \;\\ & \;F_{2}^{L}\left(k,\;\delta \right)=F_{3}^{L}\left(k,\;\delta \right) \end{array}$$

The less than alternative for pairwise Wilcoxon tests is then,

(7) $$\begin{array}{llll} & H_{a}:F_{1}^{L}\left(k,\;\delta \right)>F_{2}^{L}\left(k,\;\delta \right)\;\mathrm{and}\;F_{1}^{L}\left(k,\;\delta \right)>F_{3}^{L}\left(k,\;\delta \right)\;\mathrm{and}\; & & \;\\ & \;F_{2}^{L}\left(k,\;\delta \right)>F_{3}^{L}\left(k,\;\delta \right) \end{array}$$

More precisely, *H_a_* means that MDD censored distances tend to be smaller than Ctrl censored distances and HR censored distances tend to be smaller than Ctrl censored distances and MDD censored distances tend to be smaller than HR censored distances. The greater than alternatives are similar except that the inequalities being reversed. Then we plot *p-*values against censoring distance values. We perform similar analysis for right censored distances also. The null hypotheses for pairwise *t*-tests are similar to the ones in Eqs 6 and 7 with *F* being replaced by μ and the inequalities reversed.

**Figure 4:**
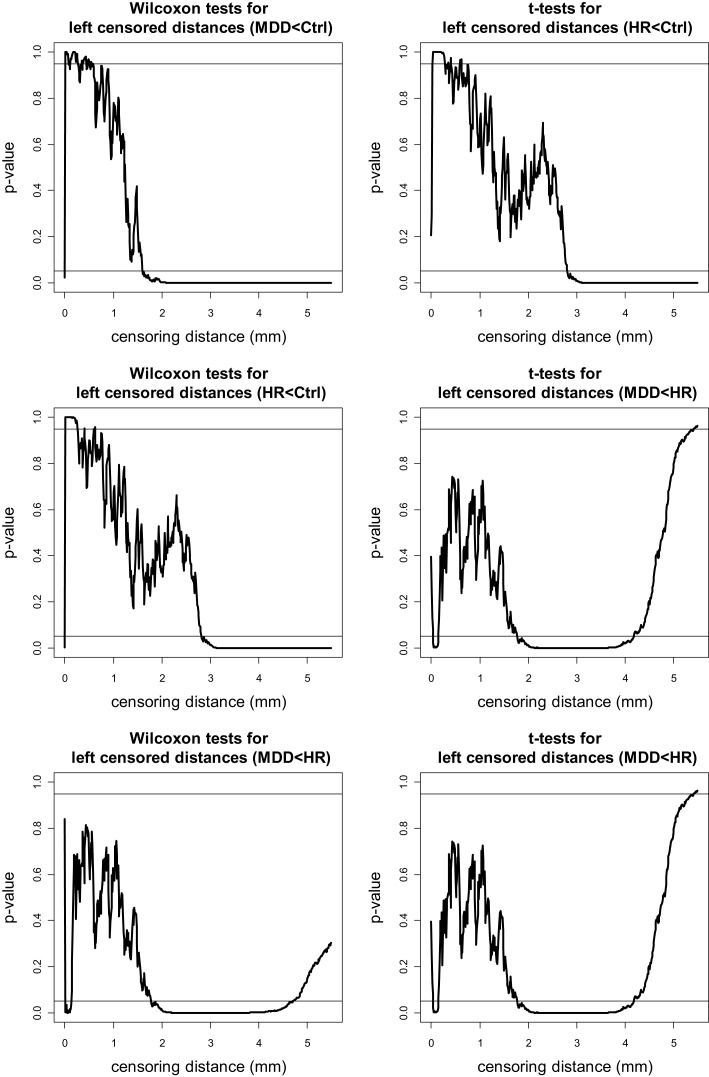
**The *p*-values versus censoring distance values (millimeter) for pairwise comparisons of left VMPFC distances with Wilcoxon rank sum test (left) and *t*-test (right) for the less than alternative**. Horizontal lines are located at 0.05 and 0.95. <: the alternative for “first less than second”; >: the alternative for “first greater than second.”

The *p-*values for left VMPFC groups are plotted in Figure 4, where the plots are for “MDD < Ctrl,” “HR < Ctrl,” and “MDD < HR” alternatives. Since the one-sided tests are complementary, in the sense that, the resulting *p*-values for the left-sided and right-sided alternatives should add up to one, we only present the “less than (<)” alternatives for the pairwise tests. Notice that at each plot, 0.05 and 0.95 are indicated by horizontal lines, and if the *p*-value falls below 0.05 (above 0.95), then the test is significant for the “less than (<)” [“greater than (>)”] alternative. Based on the plots of the *p*-values of the “less than” alternatives for left VMPFCs, we see that MDD left censored distances tend to be significantly smaller than Ctrl left censored distances of 1.6 mm or higher. That is, at distance values of 1.6 mm or larger from the GM/WM surface, there are fewer GM voxels in MDD left VMPFCs than those in Ctrl left VMPFCs. Similarly, at distance values of 2.8 mm or larger from the GM/WM surface, there are fewer GM voxels in HR left VMPFCs than those in Ctrl left VMPFCs. On the other hand, MDD left censored distances are significantly smaller than HR left censored distances for γ*_d_*(*k*, δ) values between 1.8 and 4.6 mm. Hence, there are fewer GM voxels in MDD left VMPFCs at distance values in [1.8, 4.6] mm. Based on the results of the *t*-tests (see Figure 4), we notice virtually the same results, except that mean distance for MDD left VMPFCs is significantly smaller than that of HR left VMPFCs at distances between 1.8 and 4.2, while MDD left distances tend to be smaller (in ranking) than HR distances for distances between 1.8 and 4.6 mm.

**Figure 5:**
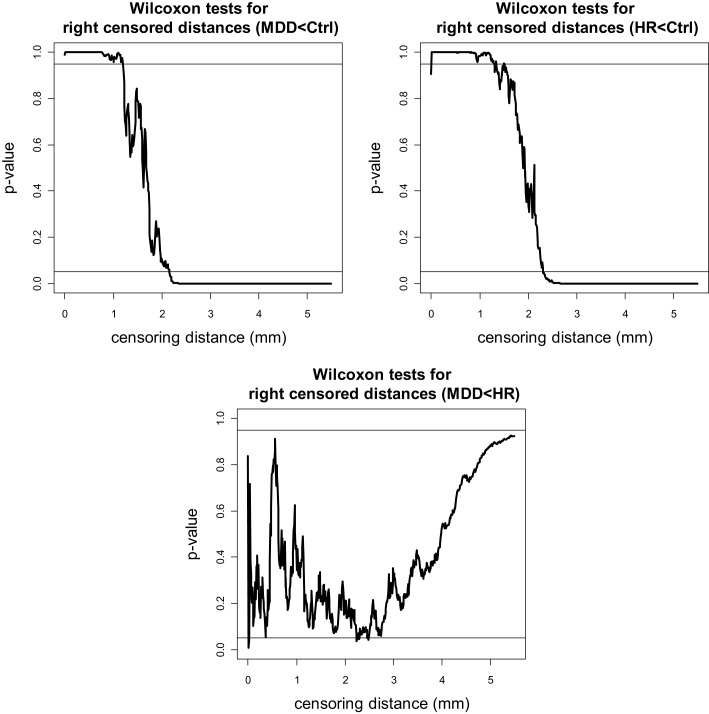
**The *p*-values versus censoring distance values (millimeter) for pairwise comparisons of right VMPFC distances with Wilcoxon rank sum test for the less than alternative**. Horizontal lines and alternatives are as in Figure 4.

The *p-*values for pairwise Wilcoxon tests for right VMPFC groups are plotted in Figure 5 (plots for pairwise *t*-tests are virtually same, hence omitted). Notice that there are fewer GM voxels in MDD right VMPFCs at distance values between 0 and 1.5 mm (of which only the range 1–1.5 mm is reliable) and at 2.1 mm or higher compared to Ctrl right VMPFCs. Similarly, there are fewer GM voxels in HR right VMPFCs at distance values between 0 and 1.5 mm (of which only the range 1–1.5 mm is reliable) and at 2.2 mm or higher compared to Ctrl right VMPFCs. On the other hand, the distances for MDD and HR right VMPFCs are not significantly different for the entire range of 0–5.5 mm, except that MDD distances are significantly smaller for distance values around 2.2 and 2.5 mm.

**Figure 6:**
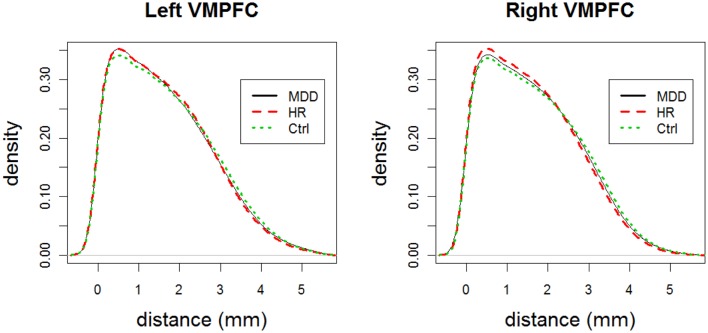
**The plots for the kernel density estimates of the pooled LCDM distances for each of MDD, HR, and Ctrl groups for left and right VMPFCs**.

**Remark 3.1: Analysis of censored LCDM distances versus distribution comparisons:** Observe that analysis of censored LCDM distances provides much more information compared to comparisons of the distributions of pooled distances. In particular, Wilcoxon test and K–S tests do not provide the distance values at which the differences occur. K–S test together with the ecdf plot might provide further details on the morphometry of VMPFCs compared to Wilcoxon test. However, ecdf plots suffer from the cumulative nature of the distances. On the other hand, kernel density estimates provide information on how frequent the voxels are at particular distance values. We present the kernel density plots of the LCDM distances for left and right VMPFCs by group in Figure 6 which suggests that smaller distances are more frequent (with respect to the total number of GM voxels for both groups) for MDD and HR VMPFCs. Furthermore, these density plots suggest that MDD and HR distances are more similar (up to, maybe, a scale factor) compared to the Ctrl distances. Observe also that the kernel density estimates agree with the results of the censored distance results plotted in Figures 4 and 5. However, although kernel density plots and censored LCDM distance analysis provide similar information, we cannot assign statistical significance to the differences by just using the kernel density estimates. ■

**Remark 3.2: Effect of the bin size on censored LCDM distances:** The sensitivity of the censored distances depend on the bin size, δ. We have experimented with various resolutions in the bin width for the censoring (not presented). Since the GM/WM surface has a complicated structure, our partitioning of the ROI with voxels yields very different distance measures, i.e., not as multiples of the voxel resolution (recall that LCDM distances are from the GM/WM surface to the center of mass of a voxel; and voxel partitioning is not performed as parallel layers from the GM/WM surface). The distances should be measured with more decimal places than the bin size in order not to make many censoring steps redundant (and here we have distances up to five decimal places). Here, decreasing the bin width further would only increase the time of the analysis, but not any qualitative difference in our results. On the other hand, increasing the bin size potentially may conceal some of the information. For example, the plots would be more like step-wise function plots, and we would only know at which interval the differences are occurring instead of its actual distance value. Thus, the bin sizes larger than the voxel resolution, *h*, will potentially conceal the local differences, and smaller than the decimal precision of the distances will only result in the computational time (with virtually no gain). So, in general, we recommend bin size, δ, to be at the precision of the LCDM distances up to at most the voxel resolution, *h*. That is, we suggest the use of bin size between 0.01 and 0.5 mm here, since if too large, censored distances do not provide the desired resolution in the distances from the GM/WM surface; and if too small, censored distances do not improve on the results of 0.01 mm. Hence the lower limit on the bin size is only due to practical concerns. ■

**Remark 3.3: Holm’s correction for simultaneous pairwise comparisons:** At each censoring distance step, we have also performed an adjustment to the *p*-values obtained from Wilcoxon rank sum tests (or *t*-tests) for simultaneous pairwise comparisons by Holm’s correction method (40). However, the adjusted and unadjusted *p*-value graphs and hence the results were similar, hence we did not present the adjusted the *p*-values for simultaneous comparisons (at the group level). Furthermore, after the Holm’s correction is applied for simultaneous multiple comparisons for each alternative, the resulting *p*-values are modified, hence not complementary to each other. Hence we would need separate plots for the “less than” and “greater than” alternatives. So our choice of omitting this adjustment is only for convenience in presentation and when simultaneous group-wise comparisons are of interest, such a correction should be employed. Also, we do not perform an adjustment for the comparison of the groups for the censoring steps, since it is the consecutive list of distances that significance persists that is more important, rather than the simultaneous comparisons, as is common practice in spatial data analysis [e.g., for Ripley’s K-function (41)]. Additionally, the analyses at the censoring steps are not independent (in fact they are highly dependent). Furthermore, there is a tendency that a test result at a censoring step is similar to the result in the preceding step. In this regard, our approach is similar to Ripley’s K-function or its variants, where the number of events or objects are counted and plotted against its expected value at increasing distance values with no adjustment for multiple testing. In both cases (censoring or Ripley’s type methods), the goal is not simultaneous inference for all the distances considered, but the trends in the differences between groups with increasing distance. Also, in either case, although inference is performed for a range of distance values, it is implicitly understood that the tests and the analysis is done in a pointwise sense at each distance value. Moreover, our simulation study suggests that multiple comparison adjustment between the censoring steps is not actually needed, as without such an adjustment the method indicates the actual distances at which significant differences occur. ■

### The influence of aggregation of censored distances and assumption violations on the tests: A Monte Carlo study

We have investigated the influence of the assumption violations on the tests due to the spatial correlation and non-Gaussianity inherent in the pooled LCDM distances and demonstrated that influence of such violations is negligible (10). In this section we demonstrate that censored distances inherit this robustness – to assumption violations – of the pooled distances as well, since the censoring procedure is applied on the pooled distances. When censoring LCDM distances, at each step, the distances accumulate, which might confound the tests and their sensitivity to detect the differences between the groups. Furthermore, at each censoring step, the dependence of LCDM distances for each person persists, and distances are significantly non-Gaussian. Here we investigate the confounding influence of such accumulation and assumption violations by Monte Carlo simulations. The most crucial step in the Monte Carlo simulation is generating distances resembling (i.e., simulating the true randomness in) LCDM distances of GM tissue in VMPFCs. For completeness, we provide the distance generation procedure, which was also described (10).

#### Simulation of realistic LCDM distances

We choose the left VMPFC of HR subject 1 (called reference VMPFC henceforth) for illustrative purposes. The LCDM distances for the reference VMPFC are denoted as $D_{21}^{L}$. We partition the distances, $D_{21}^{L}$, so that first stack of distances are in the interval *I*_0_: = [−1, 0.5] mm, the second stack of distances are in *I*_1_: = (0.5, 1.0] mm, the third stack of distances are in *I*_2_: = (1.0, 1.5] mm, and so on until the last stack of distances are in *I*_11_: = (5.5, 6.0] mm. Let ν*_i_* be the number of voxels whose distances fall in *I_i_*, i.e., $\nu_{i}=\left|D_{21}^{L}⋂I_{i}\right|$, for *i* = 0, 1, 2, …, 11. We generate *n* numbers in {0, 1, 2, …, 11} independently with the discrete probability mass function *P_N_*(*N_j_* = *i*) = ν_i_/11659 for *i* = 1, 2, …, 11 and *j* = 1, 2, …, *n*. So, *P_N_*(*N_j_* = *i*) = ν*_p_*_,_*_i_* where,

(8) $$\begin{array}{l} {\vec{\nu }}_{p}=(\nu_{p,\;0},\;\nu_{p,\;1},\ldots ,\;\nu_{p,11})=(0.177,\;0.163,\;0.151,\;0.143,\\ \;0.126,\;0.109,\;0.070,\;0.036,\;0.012,\;0.007,\;0.005,\;0.001). \end{array}$$

Let *n_i_* be the frequency of *i* among the *n* generated numbers from {0, 1, 2, …, 11} with distribution *P*_0_ for *i* = 1, 2, …, 11. Hence $n=\sum_{i=0}^{11}n_{i}$. Then we generate *U_ik_* ∼ Unif(0, 1) for *k* = 1, 2, …, *n_i_* for each *i*. Then we divide each distance by two to scale the range of generated distances to [0, 6.0] mm which is roughly the range of $D_{21}^{L}$, so the generated distances are *d_ik_* = (*i* + *U_ik_*)/2. Hence the set of simulated distances is,

(9) $$\begin{array}{llll} & D_{\mathrm{mc}}=\left\{d_{ik}=\left(i+U_{i}\right)∕2\;:U_{i}\overset{iid}{∼}\;\mathrm{Unif}\;\left(0,1\right)\;\mathrm{for}\right.\; & & \;\\ & \;\left.i=0,\;1,\;2,\;\ldots \;,\;N_{i}\;\mathrm{and}\;N_{i}∼P_{N}\;\mathrm{for}\;i=0,\;1,\;2,\;\ldots \;,\;11\;\;\;\;\right\}. \end{array}$$

The Monte Carlo scheme described above generates distances that resemble LCDM distances for the reference VMPFC. Therefore, the distances generated in this fashion together with modification of some parameters such as ν*_p_*_,_*_i_* would resemble the distances of VMPFCs from real subjects (10).

We generate three samples X, Y, and Z each of size *n_x_*, *n_y_*, and *n_z_*, respectively in our Monte Carlo simulations with *n_x_* = *n_y_* = *n_z_* = 10000 as below. For example, we generate sample Xas follows. Let η*_x_* be a positive integer less than the maximum number of voxels in the stacks in Eq. 8, namely 2059 and $\nu_{x}=\left(\nu_{0}^{x},\;\nu_{1}^{x},\;\ldots \;,\;\nu_{12}^{x}\right)$ with $\nu_{i}^{x}$ being the *i*th entry in ***ν****_x_* such that $\nu_{i}^{x}$ is the *i*th value after the values |ν*_i_* − η*_x_*| are sorted in descending order for *i* = 0, 1, 2, …, 11 and $\nu_{12}^{x}=11659-\sum_{i=0}^{11}|\nu_{i}-\eta_{x}|$. Then we generate $N_{X}=\left\{J∼P_{X},J=1,\;\ldots \;,\;n_{x}\right\}$, where $P_{X}\left(J=i\right)=\nu_{i}^{x}∕\sum_{i=0}^{12}\nu_{i}^{x}\;$. Furthermore, let $n_{i}^{x}$ be the frequency of *i* among the *n_x_* generated numbers from *P_X_*. Then we generate *U_ik_* ∼ Unif(0, *r_x_*) for $k=1,\ldots ,n_{i}^{x}$ for each *i*, where *r_x_* is a positive real number <2. Equivalently, the set of simulated distances for set X is,

(10) $$\begin{array}{llll} & D_{\mathrm{mc}}^{X}=\left\{\left(J_{i}+U_{i}\right)∕2\;:J_{i}\overset{iid}{∼}\;P_{0}\;\mathrm{and}\;U_{i}\overset{iid}{∼}\;U\;\left(0,1\right)\;\mathrm{and}\;J_{i}\;\mathrm{and}\right.\; & & \;\\ & \left.\;U_{i}\;\mathrm{are}\;\mathrm{independent}\;\mathrm{for}\;i=0,\;1,\;2,\;\ldots \;,\;n_{x}\right\} \end{array}$$

Notice that the parameters that determine the set of distances are η*_x_* and *r_x_* with η*_x_* = 0 and *r_x_* = 1, we have distances similar to our initial choice of the reference VMPFC. Moreover, as η*_x_* gets larger, the distances tend to have larger values compared to the reference VMPFC, and as *r_x_* gets larger the distances tend to have more different rankings and accumulation around *k*(*r_x_* − 1) for *k* = 1, 2, …, 11. We generate samples Y and Z in a similar fashion with parameters η*_y_*, *r_y_*, and η*_z_*, *r_z_*, respectively.

#### Empirical size curves

Under the null hypothesis of multi-sample case *H_o_*: “equality of the distributions of LCDM distances,” we generate three samples X, Y, and Z with the below parameters:

(11) $$H_{o}:r_{x}=r_{y}=r_{z}=1.0\;\mathrm{and}\;\eta_{x}=\eta_{y}=\eta_{z}=0$$

Observe that each sample of X, Y, and Z is generated so as to resemble the reference VMPFC. The choice of the reference VMPFC is done without loss of generality, since any other VMPFC can either be obtained by a rescaling the distances and/or modifying the parameters.

The censoring of the X distances is applied as in Section “Data Description and Acquisition” and the censored distances are denoted as,

(12) $$\;C_{d}^{X}\left(k,\delta \right):=\left\{\;d\in D_{\mathrm{mc}}^{X}⋂\left[0,\;k\delta \right]\;\right\}=\left\{\;d\in D_{\mathrm{mc}}^{X}:d\leq k\delta \;\right\}.$$

Samples Y and Z are generated similarly with generated distances are denoted as $D_{\mathrm{mc}}^{Y}$ and $D_{\mathrm{mc}}^{Z}$ and censored distances are denoted as $C_{d}^{Y}\left(k,\delta \right)$ and $C_{d}^{Z}\left(k,\delta \right)$, respectively.

**Figure 7:**
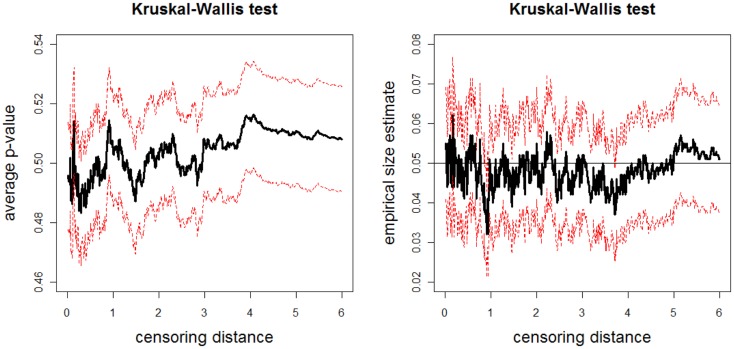
**Plotted in the left is the average *p*-values (solid line) and in the right is the empirical size estimates (solid line) versus censoring distance values for multi-group K–W test together with the 95% confidence bands (dashed lines) based on 1000 Monte Carlo replications under the null hypothesis in expression Eq. 11 which implies distributional equality of censored X, Y, and Zvalues**. Horizontal line in the right plot is at 0.05.

**Figure 8:**
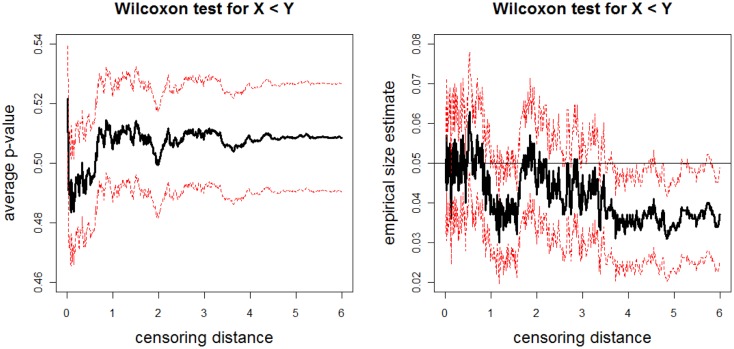
**The average *p*-values (left) and empirical size estimates (right) versus censoring distance values for pairwise Wilcoxon rank sum test for the left-sided alternative *X* < *Z***. The average *p*-values and empirical sizes (solid lines) together with the 95% confidence bands (dashed lines) are based on 1000 Monte Carlo replications under the null hypothesis of distributional equality of censored **X** and **Z** sets that are generated as described in Section “Simulation of realistic LCDM distances”. Horizontal line in the right plot is at 0.05.

The above data generation procedure is repeated *N*_mc_ = 1000 times. At each censoring step, we record the *p*-values for K–W test, and ANOVA *F-*tests (with and without HOV), and pairwise Wilcoxon rank sum test and *t*-test. We also count the number of times the null hypothesis is rejected at α = 0.05 level for these tests, thus obtain the empirical significance levels under *H_o_* in Eq. 4. The average *p*-values and empirical size estimates together with 95% confidence bands for K–W test are plotted against the censoring distance values in Figure 7; for pairwise Wilcoxon rank sum test for the one-sided alternatives *X* < *Z* are plotted in Figure 8 (the plots for *X* > *Z* are similar, hence omitted). In the left plot in Figure 7, we only plot the horizontal line at 0.05 only, since the alternative hypothesis for K–W is not one-sided. The alternative hypothesis for Wilcoxon test can be one-sided, so, if the *p*-values are smaller than 0.05, then sample X tends to be smaller than sample Z, while if they are larger than 0.95, then sample X tends to be larger than sample Z. Observe that average *p*-values are about 0.50 and empirical sizes are about 0.05 for both tests. This implies that under the null case, as expected, the simulated distances do not reveal significant differences. The empirical sizes are about the specified nominal level of 0.05 (i.e., the test is neither conservative nor liberal in rejecting the null hypothesis). Hence, the proposed procedure generates LCDM distance sets that not only resemble the VMPFCs of the subjects, but also possess the desired randomness in distances. That is, if the morphometry of the VMPFCs (quantified by the LCDM approach) had the same distribution for a set of subjects, their LCDM distances could have looked like the generated distances. The plots for ANOVA with and without HOV, for one-sided alternatives with pairwise Wilcoxon test for pairs *X*, *Y* and *Y*, *Z*, and pairwise *t*-test for all three pairs are similar (hence not presented).

#### Empirical power curves

We consider the alternative hypotheses in which we generate sample X as in the null case, so $D_{\mathrm{mc}}^{X}$ is as in Eq 10. For sample Y, we set *r_y_* = 1.2 and η*_y_* = 0 and for sample Z we set *r_z_* = 1.0 and η*_z_* = 50. So the alternative hypothesis we consider is,

(13) $$H_{a}:r_{x}=r_{z}=1.0,\;r_{y}=1.2\text{ and }\eta_{x}=\eta_{y}=0,\;\eta_{z}=50$$

and we set *n_x_* = *n_y_* = *n_z_* = 10000. So, $P_{Y}\left(J=i\right)=\nu_{p,i}^{y}$ where $\left(\nu_{p,0}^{y},\nu_{p,1}^{y},\ldots ,\nu_{p,11}^{y}\right)=\nu_{p}^{y}$ are as in Section “The Influence of Aggregation of Censored Distances and Assumption Violations on the Tests: A Monte Carlo Study”; and $P_{Z}\left(J=i\right)=\nu_{p,i}^{z}$ where,

(14) $$\begin{array}{l} \left(\nu_{p,0}^{z},\;\nu_{p,1}^{z},\;\ldots \;,\;\nu_{p,11}^{z}\right)=\nu_{p}^{z}=(0.171,\;0.158,\;0.146,\;0.138,\\ \;0.121,\;0.104,\;0.065,\;0.051,\;0.031,\;0.008,\;0.003,\;0.003,\;0.001). \end{array}$$

**Figure 9:**
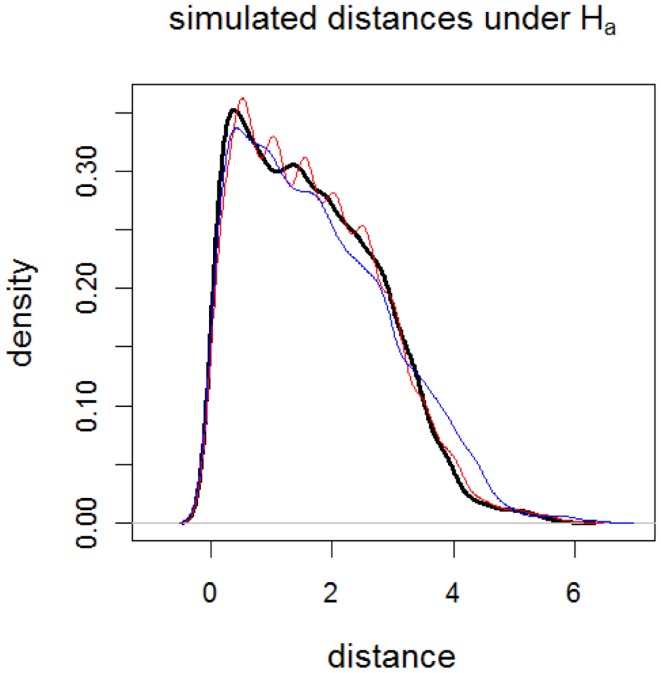
**Plots of the kernel density estimates of the Monte Carlo distances under the alternative *H_a_*:*r_x_* = 1.0, *r_y_* = 1.2, *r_z_* = 1.0, and η*_x_* = 0, η*_y_* = 0, η*_z_* = 50**. Thick solid black line is for sample **X**, thin solid red line is for sample **Y**, and thin solid blue line is for sample **Z**.

Notice that by construction sample Y is generated so that the rankings of distances are more different than those of sample X rather than the distances from the GM/WM surface. Furthermore, sample Y contains distances that are more accumulated at intervals [0.5, 0.6], [1.0, 1.1], … , [5.5, 5.6] compared to sample X. Therefore, at distances around these intervals (i.e., around γ*_k_*_,0.01_ for *k* = 50, 100, … , 550 or around γ*_k_*_,0.01_ = 0.5, 1.0, … , 5.5), the censored distances for sample X tend to be smaller than censored distances for sample Y. On the other hand, comparing $\nu_{p}^{z}$ in Eq 14 with ***ν****_p_* in Section “The Influence of Aggregation of Censored Distances and Assumption Violations on the Tests: A Monte Carlo Study,” we see that sample X is more likely to have distances <4.0 compared to those of sample Z. Hence, we expect that the censored distances for sample X to be smaller than censored distances for sample Z at γ*_k_*_,0.01_ for *k* ≥ 400 (i.e., γ*_k_*_,0.01_ ≥ 4.0). Likewise, we expect that for distances larger than 4.0, the censored distances for sample Y to be smaller than censored distances for sample Z. See Figure 9 for the kernel density estimates of sample distances under the alternative hypothesis in Eq 13, which agrees with the above discussion.

**Figure 10:**
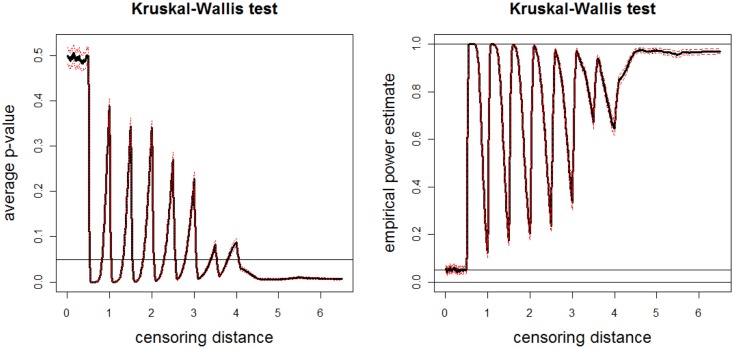
**Plotted in the left is the average *p*-values (solid line) and in the right is the empirical power estimates (solid line) versus censoring distance values for multi-group K–W test together with the 95% confidence bands (dashed lines) based on 10000 Monte Carlo replications of censored X, Y, and Z sets that are generated under the alternative hypothesis Eq 13**. Horizontal lines are at 0.05 and 0.95.

We repeat this sample generation procedure *N*_mc_ = 1000 times and estimate empirical power by counting the number of times the null hypothesis is rejected at α = 0.05. The average *p*-values and empirical power estimates together with 95% confidence bands versus censoring distance values for multi-group K–W test are plotted in Figure 10. Observe that there are significant differences between groups around γ*_k_*_,0.01_ = 0.5, 1.0, …, 3.5, and for distances larger than 4.0 as expected. The significant differences at steps of 0.5 increments is mostly because of sample Y, and for distances larger than 4.0 is mostly because of sample Z. The plots for ANOVA with or without HOV are similar (hence not presented).

**Figure 11:**
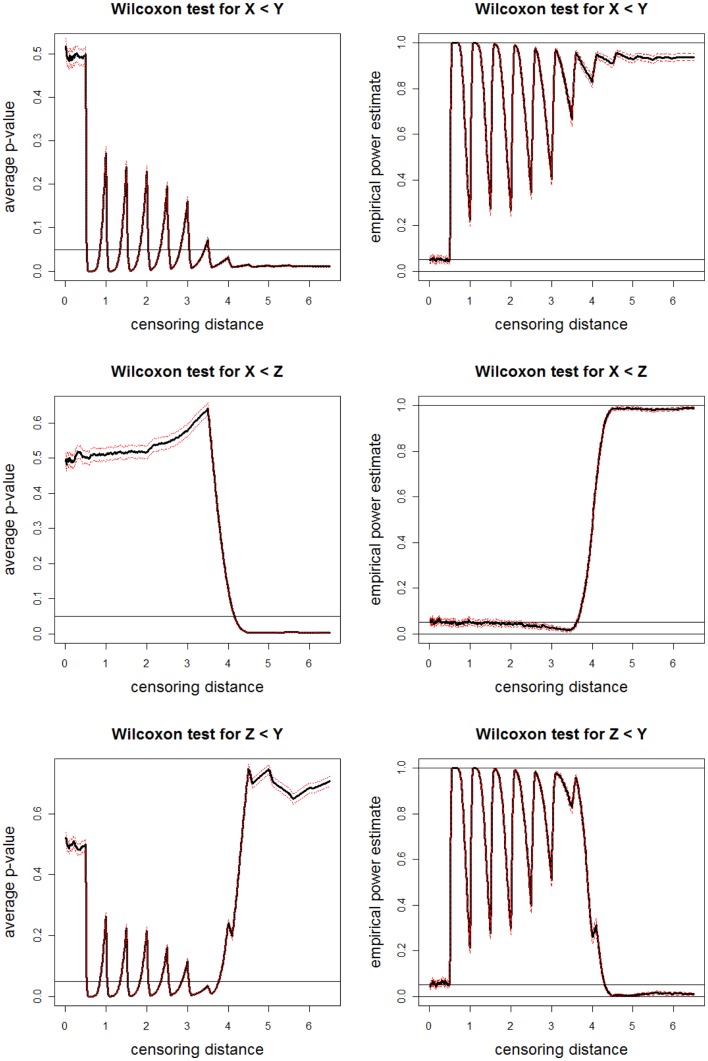
**Plotted in the left are the average *p*-values (solid lines) and in the right are empirical power estimates (solid lines) versus censoring distance values for Wilcoxon rank sum test for the left-sided alternatives *X* < *Y* (top), *X* < *Z* (middle), *Z* < *Y* (bottom) together with the 95% confidence bands (dashed lines) as in Figure 10**. Horizontal lines are at 0.05 and 0.95.

The average *p*-values and empirical power estimates together with 95% confidence bands versus censoring distance values for pairwise Wilcoxon rank sum tests for the left-sided alternatives *X* < *Y*, *X* < *Z*, and *Y* < *Z* are plotted in Figure 11. Based on pairwise Wilcoxon test for *X* < *Y* alternative, we observe that censored distances for sample X tend to be smaller than censored distances for sample Y around γ*_k_*_,0.01_ for *k* = 50, 100, …, 350 and *k* ≥ 350 (i.e., around γ*_k_*_,0.01_ = 0.5, 1.0, …, 3.5 and at γ*_k_*_,0.01_ ≥ 3.5). For censored distances larger than 4.0, the proportions are not large enough for samples X and Y to balance the accumulation of Y distances around 4.0, 4.5, 5.0, and 5.5. Hence, censored distances for sample Y are significantly larger than those of sample X for γ*_k_*_,0.01_ ≥ 3.5. Based on pairwise Wilcoxon test for *X* < *Z* alternative, we observe that censored distances for sample X tend to be smaller than censored distances for sample Z at γ*_k_*_,0.01_ for *k* ≥ 400 (i.e., at γ*_k_*_,0.01_ ≥ 4.0). For censored distances larger than 4.0, the proportions have larger weights for sample Z. Hence, censored distances for sample Z are significantly larger than those of sample X. Based on pairwise Wilcoxon test for *Z* < *Y* alternative, we observe that censored distances for sample Y tend to be larger than censored distances for sample Z around γ*_k_*_,0.01_ for *k* = 50, 100, …, 350 (i.e., around γ*_k_*_,0.01_ = 0.5, 1.0, …, 3.5). For censored distances larger than 4.0, the proportions are not large enough for sample Z to make its censored distances larger than those of sample Y. Hence, censored distances for sample Z are not significantly different from those of sample Y for γ*_k_*_,0.01_ ≥ 4.0 with virtually zero power. This also occurs because of the proportions having larger weights for distances <4.0, and any parameter affecting these distances have more influence in censored distance analysis. The results of pairwise *t*-tests are similar (hence not presented).

## Discussion

In this article, we introduce the censoring of the (pooled) LCDM distances which provide information on the laminar thickness of the cortical tissue. The summary measures such as mean, median, or variance of distances for each subject could be analyzed. However summarization of LCDM distances for each subject causes loss of information conveyed by the LCDM distance. Previously, to characterize all of the LCDM distances and to obtain an overall VMPFC for each diagnostic condition, we pool (i.e., merge) LCDM distances of the subjects in the same diagnostic group in one data set (10).

Pooled LCDM distances provide a method to analyze heterogeneous forms of morphometric differences (10). When the LCDM distances of the subjects in the same diagnostic group are pooled, the most common morphometric traits of the VMPFCs for that group are more emphasized. On the other hand, the morphometric traits not common for most subjects in a group but specific to a particular subject are downplayed. The most common morphometric traits in VMPFCs in a particular diagnostic group are more likely to be related to the diagnosis of the group and pooled LCDM distances carry on the most common characteristics, so they can be very sensitive in detecting the diagnosis-specific traits of VMPFCs. As a result, pooled LCDM distances can be suggestive of the changes in VMPFC due to a disease. When pooled distances yield significant results, it implies that VMPFCs significantly differ in morphometry (shape or size) associated with the diagnostic conditions. However, it is not suggestive of the locations of such differences, which might be important for understanding the underlying neurobiology. Hence, we propose the censoring of the pooled LCDM distances in this article to further characterize the nature of the regional differences in the specified distance with respect to the GM/WM surface due to specific diseases. When the pooled LCDM distances are censored, the locations of the most common characteristics of VMPFCs in each group are more emphasized; hence one can detect the location of the changes in VMPFC of a subject due to, say, depression. So, censored distances inherit the nice properties of the pooled distances such as the sensitivity of the pooled distances to disease specific morphometric differences. When significant results are obtained from the censored distance analysis, it provides the distance from the GM/WM surface at which cortical mantle starts to differ in morphometry. Hence compared to pooled distances, analysis of censored distances is potentially more useful for diagnostic or clinical purposes and may provide more sensitive characterization for longitudinal treatment evaluation.

We use K–W and ANOVA (with or without HOV) *F-*tests for multi-group comparisons; and Wilcoxon rank sum, and *t*-tests tests for two-group comparisons (the first of these are used to test distributional differences and the second is used to test mean differences due to a location parameter). But, all of these tests require within sample independence, which is violated due to the spatial correlation between LCDM distances of nearby voxels. However, the influence of this violation is mild or negligible for pooled distances (10). We demonstrate that analysis of censored distances is also robust to such assumption violations, by extensive Monte Carlo simulations and this is another nice property inherited by censored distances. Furthermore, the influence of aggregation of censored distances for larger censoring distances is mild to negligible. Hence we recommend both parametric and non-parametric tests for censored LCDM distances, since they are more sensitive against different alternatives. In particular, K–W and Wilcoxon tests are more sensitive to distributional differences of GM voxels at about the same distance, while ANOVA *F*-tests and *t*-tests are more sensitive against the differences in the means, that is, differences in average GM distances. One caution about censoring distances is that, major significant differences for smaller distances might confound the differences for larger distance values. However, this might be overcome by using tests on the censoring distances and K–S test together with empirical cdf plots.

As an illustrative example, we use GM tissue in VMPFCs as the ROI for three groups of subjects; namely, subjects with MDD, subjects at HR for MDD, and healthy control subjects (Ctrl). We found that there are significant morphometric differences between the groups at distances from the GM/WM surface of 2.00 mm or larger in the left VMPFC and between 1.00 and 1.20 mm and at 2.20 mm or larger in the right VMPFC. Furthermore, we see that left VMPFCs in MDD and left VMPFCs in Ctrl subjects show significant morphometric differences at distances of 1.60 mm or larger with significant reduction in left VMPFC associated with a history of major depression. Similarly, left VMPFCs in HR and Ctrl subjects are significantly different at distance values of 2.80 mm or larger with significant reduction in the left VMPFC in HR. That is, left VMPFC of MDD subjects tend to shrink more, since significant morphometric differences start to occur at 1.60 mm in MDD and 2.80 mm in HR left VMPFCs. On the other hand, left VMPFC in MDD is significantly smaller than HR at distances between 1.80 and 4.60 mm. Right VMPFCs in MDD and Ctrl subjects are significantly different at distances between 1.00 and 1.50 mm and at 2.10 mm or higher, with significant reduction in MDD. Similarly, right VMPFCs in HR and Ctrl are significantly different for distances between 1.00 and 1.50 mm and at 2.20 mm or higher. That is, in terms of distances, MDD and HR right VMPFCs tend to shrink but slightly more for MDD right VMPFCs (distance values of 2.20 mm for HR and 2.10 mm for MDD and between 1.00 and 1.50 mm for both) compared to Ctrl right VMPFCs. Right VMPFCs in MDD and HR are not significantly different for distances except around 2.20 and 2.50 mm. We thus observe a significant reduction in laminar thickness of the VMPFC and perhaps shrinkage in MDD when compared to Ctrl subjects. A similar trend can also be observed when HR is compared with the Ctrl LCDM distances. But significant morphometric differences occur at different GM distance values. These findings suggest that differences in the right VMPFC are not a consequence of episodes of MDD, but these differences are associated with higher genetic risk of MDD. Therefore, censored distances provide much more detailed information compared to pooled distances, and more powerful to help identify the local implications of the disease in the ROI.

At the microscopic level, the cortical mantle is thought to be composed of six cortical layers that are numbered I to VI as one goes from the outer or pial, i.e., GM/CSF boundary away from the skull inwards to the GM/WM surface (42). Each layer is thought to comprise of different cells such as neuronal, pyramidal, non-pyramidal, and glial cells that are important in neurotransmission between the different layers as well as with other cortical and subcortical regions (42). Estimates of neuronal and glial densities in different cortical regions have been obtained from several histopathological, i.e., post-mortem studies in humans and mammals. Reduced measures have been suggested as plausible explanations for cortical thinning observed in several neuroimaging studies albeit at the macroscopic level [e.g., Ref. (43)]. In particular, in a histopathological study of major depression in humans, (44) showed both reduction in neuronal and glial density in subregions of the prefrontal cortex but that reduction in glial density was unique in the dorsolateral prefrontal cortex. Specifically they showed differences in densities in the upper cortical layers (II–IV) i.e., at distances far from the GM/WM surface or equivalently close to the pial surface in the rostral orbitofrontal regions and in the lower cortical layers (V–VI) i.e., at distances close to the GM/WM surface in the caudal orbitofrontal regions. Differences have also been demonstrated in a subportion of the VMPFC, the subgenual prefrontal cortex (44, 45). While no histopathological studies of the overall VMPFC have been done, it is conceivable that differences in censored LCDMs at distances from the GM/WM surface may be characterized by corresponding density changes. However, no definitive conclusion can be reached until LCDM analysis of a specific cortical region can be correlated with histopathological measures in an animal model of a neuropsychiatric disorder.

Labeled cortical distance mapping analysis could be used and tested further. In particular, the suggested methodology can be useful in the following scenarios: (a) with higher-resolution scanners, an increased voxel resolution (say <1 mm) would be obtained and the methodology can be adapted for performing detailed analysis within the GM of the cortex (46); (b) classification purposes where predictive sensitivity is more important than the specificity where different measures of the cortical structure can be learned via tools based on LCDM methodology.

In summary, we have shown how LCDM distances can be used to estimate the location of differences in the cortical mantle (in terms of distance from the GM/WM surface), if censoring is performed after pooling. Such an approach can be used to analyze other cortical structures implicated in various neuropsychiatric and neuro-developmental disorders.

## Author Contributions

Elvan Ceyhan developed the methodology, analyzed the data and wrote the manuscript. Tomoyuki Nishino and Dimitrios Alexopolous acquired and processed the data. Richard D. Todd, Michael I. Miller, Kelly N. Botteron, and J. Tilak Ratnanather developed the scientific overview. In addition Kelly N. Botteron and J. Tilak Ratnanather assisted Elvan Ceyhan in writing the paper.

## Conflict of Interest Statement

The authors declare that the research was conducted in the absence of any commercial or financial relationships that could be construed as a potential conflict of interest.

## Abbreviations

CA, computational anatomy; CSF, cerebrospinal fluid; Ctrl, healthy control; GM, gray matter; HOV, homogeneity of variances; HR, high risk; K–S, Kolmogorov–Smirnov; K–W, Kruskal–Wallis; LCDM, labeled cortical distance mapping; MDD, major depressive disorder; MRI, magnetic resonance imaging; ROI, region of interest; VBCT, voxel-based cortical thickness; VMPFC, ventral medial prefrontal cortex; WM, white matter.
